# Intelligent Sensors for POI Recommendation Model Using Deep Learning in Location-Based Social Network Big Data

**DOI:** 10.3390/s23020850

**Published:** 2023-01-11

**Authors:** Wanjun Chang, Dong Sun, Qidong Du

**Affiliations:** 1 College of Computer Science & Technology, Henan Institute of Technology, Xinxiang 453003, China; 2 Educational Technology Center, Guangzhou Railway Polytechnic, Guangzhou 510430, China

**Keywords:** intelligent sensors, social network, big data, deep learning, recommended model, geographical-spatiotemporal gated recurrent unit network, points of interest

## Abstract

Aiming at the problem that the existing Point of Interest (POI) recommendation model in social network big data is difficult to extract deep feature information, a POI recommendation model based on deep learning in social networks and big data is proposed in this article. The input data are all gathered through intelligent sensors to apply some raw data pre-processing tasks and thus reduce the computational burden on the model. First, a POI static feature extraction method based on symmetric matrix decomposition is designed to capture the geographical location and POI category features in Location-Based Social Networking (LBSN). Second, the improved Continuous Bags-of-Words (CBOW) model is used to extract the semantic features in the user comment information, and realize the implicit vector representation of POI in geographic, category, semantic and temporal feature space. Finally, by adaptively selecting relevant check-in activities from the check-in history to learn and change user preferences, the Geographical-Spatiotemporal Gated Recurrent Unit Network (GSGRUN) can distinguish the user preferences of different check-in. Experiments show that when the length of the recommendation list is 15, the precision of the proposed algorithm on the loc-Gowalla data set is 0.0686, the recall is 0.0769, and the precision on the loc-Brightkite data set is 0.0659, the recall is 0.0835, both of which are better than the comparative recommendation methods. Therefore, compared with the comparison methods, the proposed method can significantly improve the performance of the POI recommendation system.

## 1. Introduction

In recent years, with the rapid development of intelligent mobile devices and information technology, revolutionary changes have taken place in the means, thinking, and mode of information acquisition, making Location-Based Social Network (LBSN) widely used [1,2,3] and becoming an important part of people’s daily life. Unlike traditional online social networks, Location-Based Social Networking (LBSN) adds real geographical location information and user comment information to social networks, thus combining users’ online behaviors with offline activities, providing a new entry point for studying users’ personalized preferences and behavior patterns [4,5,6]. In LBSN, users share Points of Interest (POI) in the form of “check-in”, and score and comment on the POI to indicate the user’s preference for the POI. At present, typical LBSN applications such as Qunar strategy, Meituan review, Dianping review, and foreign Foursquare, Gowalla, Yelp, etc. will analyze the user’s behavior habits according to the user’s geographical location and check-in information, so as to recommend the POI that users are interested in. Unfortunately, most of the existing methods in the literature which will be explained later mine shallow information in feature extraction, while a large amount of deeper feature information is difficult to extract, often resulting in low accuracy. This would result in low precision and accuracy in the model and would limit the model capabilities severely. Even the most powerful AI-based models would fall short in the face of insufficient feature selection processes. Therefore, it is critical and significant to address this deficiency. To this end, a POI recommendation model using deep learning in LBSN big data is proposed in this article.

POI personalized recommendation service is a very important research topic in the field of recommendation systems and intelligent perception. Its main task is to recommend a POI that meets the user’s personalized preferences according to a series of information with user behavior tags, such as the user’s geographic location, social relations, and check-in data [7]. POI personalized recommendation can not only help users find attractive POI in massive contextual information and meet their personalized needs, but also excavate user behavior habits for merchants, thus bringing huge economic benefits to merchants [8,9,10]. At the same time, the large-scale user-POI interaction data in LBSN also enables the industrial and academic circles to have a deeper understanding of user preferences and user behaviors. However, in practical applications, the user’s check-in record is closely related to such factors as time, geographical location, social relations, and comment information. These factors come from different fields and have different composition structures and expression methods, which have different degrees of impact on POI recommendation [11,12,13]. Therefore, how to mine the potential preferences of users according to various heterogeneous types of context information, integrate time, geography, and user’s personal preferences into the POI recommendation model, and generate a POI recommendation list that meets the user’s personalized preferences, is the difficult content in the research of personalized recommendation service of POI.

POI recommendation has become a research focus. It mainly mines the user’s location preference by analyzing the user’s historical location in check-in records and then accurately recommends the places that may be of interest to the user [14,15,16,17]. Due to the limited number of locations checked in by a single user and the large number of POIs, the data on user-POI interaction recommended by POI is very sparse. Second, most of the information collected in the LBSN is the user’s check-in records, but the user rarely leaves the scoring data when checking in at the location. The scoring data can clearly indicate the user’s preference. For example, a high score means like, whereas a low score means dislike. However, the user’s check-in record only indicates that the user has been to this place and does not indicate that the user likes this place. Perhaps the user happens to pass by this place, or the user comes to this place but does not like it. These have brought great challenges to POI recommendations [18,19,20]. Therefore, this article proposes a POI recommendation model using deep learning in LBSN big data. The proposed method would provide a richer feature extraction model based on intelligent sensors and deep learning capabilities. To summarize, the innovations of the proposed method are:

(1) A POI static feature extraction method based on symmetric matrix decomposition is designed to capture the geographical location and POI category features in LBSN. The improved CBOW model is used to extract the semantic features in the user comment information. This method realizes the implicit vector representation of POI in geographic, category, semantic, and temporal feature spaces and significantly improves the feature extraction ability of the system.
(2) By adaptively selecting relevant check-in activities from the check-in history to learn and change user preferences, the Geographical-Spatiotemporal Gated Recurrent Unit Network (GSGRUN) is used to distinguish the user preferences of different check-in and improve the accuracy of recommendation.

## 2. Related Works

The method of deep learning is based on a large amount of data to train the model and mine the potential information from the data. Therefore, deep learning can better extract features and learn high-order interactions between features. In the face of feature engineering construction on unstructured data sets, deep learning has great advantages in feature selection and high-level semantic feature learning. Reference [21] used Recurrent Neural Network (RNN) to realize personalized POI recommendations to the user according to the user’s check-in order. Reference [22] proposed a Content-Aware hierarchical POI Embedding (CAPE) model for POI recommendation based on the characteristics of POI or the relationship between POIs. Reference [23] proposed a personalized recommendation method based on a deep interest network by capturing the dynamic characteristics behind user behavior. Reference [24] believed that the effective use of temporal and spatial context information can help predict the trend of users’ check-in preference for POI and effectively improve the prediction ability of the next POI. By expanding the recurrent neural network, a Spatio-Temporal Recurrent Neural Network (ST-RNN) recommendation model was proposed. Reference [25] combined Content-Based Filtering (CBF) and Network-based Collaborative Filtering (NCF), and proposed a deep-learning-based collaborative filtering method DeepCCF for personalized resource recommendation. Reference [26] proposed a deep neural model based on transfer learning, which integrated cross-domain knowledge to achieve more accurate POI recommendations. Using deep transfer learning, this method learned the complex user-project interaction relationship and more accurately captured the overall preferences of users for transferring. Reference [27] proposed a personalized recommendation method based on the Seq2Seq model of the LSTM recurrent neural network by combining personalized recommendation and collaborative filtering recommendation methods. Reference [28] combined the original collaborative filtering matrix based on the user with the personalized preference matrix of the user and proposed a personalized collaborative fusion algorithm to make the recommendation more accurate and attractive. In order to fuse multi-source data in a unified metric space, reference [29] constructed a topic package as a metric space. It used LSTM-based methods for personalized travel route recommendations.

## 3. Proposed Model

### 3.1. Model Framework

**Figure 1:**
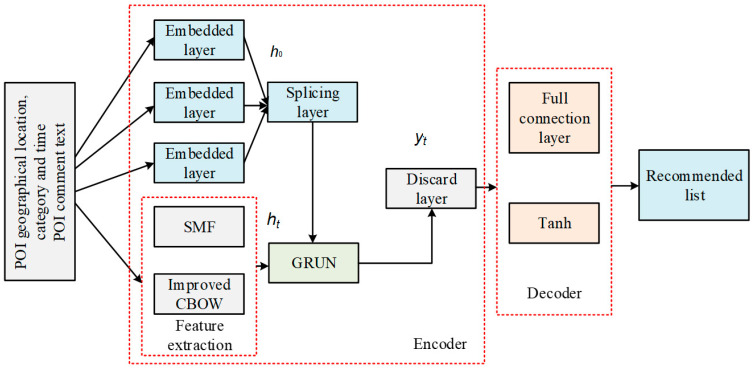
Overall framework of the proposed model.

In order to accurately extract and express POI features and improve the accuracy of POI recommendation, this paper proposes a POI recommendation model using deep learning in LBSN big data. The model uses an encoder-decoder structure in which POI feature vectors are taken as the inputs and processed by GRUN to obtain the hidden vector representation of each feature. All the input features are gathered by intelligent sensing devices to make sure that some pre-processing tasks are handled by then. In such sensors, advanced signal processing methods can help to purify the data before usage in the deep learning model. This is a big help in removing the high computational burden of data preprocessing needed by AI methods. Some examples of such sensors can be an intelligent pressure sensor, intelligent image sensor, intelligent wireless sensor, intelligent position sensor, etc. In the decoder, the fully connected layer and the nonlinear activation function are used to learn the high-order and nonlinear interaction between POI hidden feature vectors so as to realize the personalized recommendation of POI for users in specific scenarios. The overall framework of the model is shown in Figure 1. The input generated by the current POI check-in sequence includes POI geographic location context, POI category information, POI time context, and comment text in the POI check-in behavior. Additionally, *h*_0_ represents the initial hidden state, *h_t_* represents the hidden vector representation of POI features obtained by the encoder, and *ŷ_t_* is the score prediction of all POIs obtained by the model.

### 3.2. POI Feature Extraction and Representation Method

(1) POI Static Feature Extraction.

This section explains the process of feature extraction in the POI [3,4,5,6]. For a specific POI point, a geographic adjacency matrix *G* is defined. The initial value of the matrix is set to 0 and the size is the number of POIs. First, the geographic distance of any two POIs *P_i_* and *P_j_* are calculated according to the longitude and latitude of the POI, and it is written into the corresponding element in the geographic adjacency matrix *G*. Then, a distance threshold of *m* is set. The symbol *m* shows the threshold value such that when the value of the element in the *G* matrix is less than *m*, its value in the *G* matrix is set to 1, showing that the *P_i_* and *P_j_* are adjacent to each other in geographical location and thus have geographical relevance; otherwise, the value in *G* matrix becomes 0, indicating that *P_i_* and *P_j_* are not geographically related. Theoretically, the default value for the threshold is 0.5 for normalized predicted probabilities or scores but it can vary according to the operators’ experiences. It is clear that the higher value that *m* takes, the higher probability is assigned to the proximity of *P_i_* and *P_j_* and vice versa. It should be noted that the values on the diagonal of the matrix *G* represent the distance from the POI to itself, which are all less than 1, so the values on the diagonal of the matrix *G* are all 1.

The category association matrix is used to establish associations between POIs of the same category, which is helpful to alleviate the data sparsity problem in POI recommendation. Define the category association matrix *C*, set the initial value of the matrix to 0, and specify that each POI in the POI set only belongs to one specific category. If the categories of any two POIs *P_i_* and *P_j_* are the same, set the corresponding elements in the category association matrix *C* to 1, otherwise set them to 0.

According to the core idea of symmetric matrix decomposition, the POI geographical adjacency matrix is decomposed into a low-rank matrix multiplied by its transposed matrix. The specific decomposition form is:

(1) $$L=\underset{E_{g}}{\min}{\left\|G-E_{g}E_{g}^{T}\right\|}_{F}^{2}+\lambda_{g}{\left\|E_{g}\right\|}_{F}^{2}$$

where ${\left\|\cdot \right\|}_{F}^{2}$ is the Frobenius norm of the matrix, *E_g_* is the potential geographical matrix after the decomposition of the symmetric matrix, *d* is the dimension of the hidden vector, and $\lambda_{g}{\left\|E_{g}\right\|}_{F}^{2}$ is the regularization term, so that the difference between the geographical adjacency matrix *G* and $E_{g}E_{g}^{T}$ are as small as possible. The stochastic gradient descent method is used to update the model parameters, and the partial derivatives and local optimal solutions of the matrix *E_g_* are obtained.

The essence of decomposing the POI geographical adjacency matrix is to learn the similarity of POI in geographical location. The closer the geographical location of two POIs, the higher the degree of association of the two POIs, that is, the more similar the hidden vectors in the geographic feature space. If the implicit vectors of the geographical adjacency matrix of the POI *i* and *j* are represented by *e_i_* and *e_j_*, the approximate similarity of the implicit vector *e_i_* and *e_j_* is expressed by the cosine similarity as follows:

(2) $$sim(e_{i},e_{j})=\frac{\langle e_{i},e_{j}\rangle }{\sqrt{\langle e_{i},e_{i}\rangle \langle e_{j},e_{j}\rangle }}\approx G_{ij}^{’}$$

where, 〈·,·〉 represents the inner product of two vectors. The closer the geographical location of two POIs (that is, the larger $G_{ij}^{\prime }$), the higher the similarity $sim(e_{i},e_{j})$ of their hidden vectors in the feature space. This shows that the method based on symmetric matrix decomposition in this paper can learn the mutual relationship of POIs in the geographical location feature space.

Similar to the decomposition of the POI geographical adjacency matrix, the decomposition form of the POI category incidence matrix is:

(3) $$L=\underset{E_{c}}{\min}{\left\|C-E_{c}E_{c}^{T}\right\|}_{F}^{2}+\lambda_{c}{\left\|E_{c}\right\|}_{F}^{2}$$

where, *E_c_* is the potential category matrix after the decomposition of the symmetric matrix, and $\lambda_{c}{\left\|E_{c}\right\|}_{F}^{2}$ is the regularization term. The stochastic gradient descent method is used to update the model parameters. The partial derivatives and local optimal solutions of the matrix *E_c_* are obtained.

If the implicit vectors of the category incidence matrix of the POI *i* and *j* are represented by *c_i_* and *c_j_*, the approximate similarity of the implicit vector *c_i_* and *c_j_* is expressed by the cosine similarity as follows:

(4) $$sim(c_{i},c_{j})=\frac{\langle c_{i},c_{j}\rangle }{\sqrt{\langle c_{i},c_{i}\rangle \langle c_{j},c_{j}\rangle }}\approx C_{ij}^{\prime }$$

Compared with other feature representation methods such as one-hot coding, the symmetric matrix decomposition POI static feature has obvious advantages. First, the symmetric matrix decomposition can reduce the dimension of the matrix through low-rank approximation so as to effectively utilize the high-dimensional features. Second, the symmetric matrix decomposition can capture the potential relationship between the static features of the POIs by optimizing the vector representation of the static features of the POIs.

(2) POI Semantic Feature Extraction.

This paper presents a POI semantic feature extraction method based on an improved CBOW model, which can be used to quickly and effectively train word vectors and accurately obtain the potential emotional expression of users. First, TF-IDF (Term Frequency-Inverse Document Frequency) model is used to give different weights to word vectors according to the occurrence frequency of word vectors, and a word weight matrix is constructed. Then, the user comment text word set, the word weight matrix, and the introduced user emotion word set are taken as the inputs of the CBOW model. The vectors of the input layer are accumulated through the projection layer to build a Huffman tree, which is embedded into the CBOW model. Then, the word vector is continuously iterated to generate the POI semantic feature vector.

User comment text word set is $D=\left\{\left(d_{1}\right),\left(d_{2}\right),\ldots ,\left(d_{k}\right)\right\}$, word vector matrix is $F=\left\{\left(t_{1}\right),\left(t_{2}\right),\ldots ,\left(t_{s}\right)\right\}$ and user emotion word set is $H=\left\{\left(g_{1}\right),\left(g_{2}\right),\ldots ,\left(g_{m}\right)\right\}$. The user comment text word set *S* can be obtained by simply preprocessing the comment text. The word vector matrix first needs to use the TF-IDF model to process the user’s comment text and give different weights to the words according to the different frequencies of the words, so as to build a word weight matrix. The TF-IDF weight calculation formula of words *t_i_* is as follows:

(5) $$t_{i}=\frac{n_{i,j}}{\sum_{k}n_{k,j}}\times \log\frac{\left|D\right|}{\left|\{j:t_{i}\in d_{j}\}+1\right|}$$

Here, *D* shows the user comment text word, *d_j_* shows the *i*th element of set *D*, it shows the weight value assigned the *i*th word and *n_i,j_* shows the number of *i*th word in *j*th user text set.

When calculating the weight value of a word *t_i_*, there will be a phenomenon that one word corresponds to the multiple weights. This phenomenon will cause that Huffman tree cannot be built in CBOW model, thus affecting the representation of POI semantic features. Therefore, this paper uses the method of taking the average value of the weights of *t_i_* to get the unique TF-IDF weight value. The average value of the weight is $w_{a}=\sum_{i}t_{i}/\left\{j:t_{i}\in d_{j}\right\}$.

The objective function of CBOW model is:

(6) $$f(w,j,\alpha ,b_{l})=\sum_{w}\log\prod_{j=2}^{l^{w}}p(d_{j}^{w}|x_{w},\theta_{j-1}^{w})+\sum_{t}P(g_{j}^{}|w_{t};W,\alpha ,b_{l})$$

where $d_{j}^{w}$ represents the *j* position of Huffman tree encoding, *l^w^* represents the number of nodes in the path from the root node to the word *w*, and $\theta_{j-1}^{w}$ represents the auxiliary vector. Additionally, α is a setting parameter determining the balance in the model.

(3) POI Semantic Feature Extraction.

The user’s check-in on the POI is time sensitive and will change dynamically with time. For the time features ${\tilde{t}}_{t}$ of POI, the time series division method in this paper is to divide a week into 7 days and a day into 24 equal time slots. After the static, semantic, and temporal features of POI obtained through the above processing, the extracted feature vectors are concatenated to obtain the feature representation of POI. Specifically, *e_t_*, *c_t_*, *v_t_* , and ${\tilde{t}}_{t}$ ,respectively, represent the hidden vectors of the POI in the geographic, category, semantic, and temporal feature space, then the feature vectors of the POI at the current time *t* can be expressed as:

(7) $$q_{t}=[e_{t}\;c_{t}\;v_{t}\;{\tilde{t}}_{t}]$$

### 3.3. Model Training

**Figure 2:**
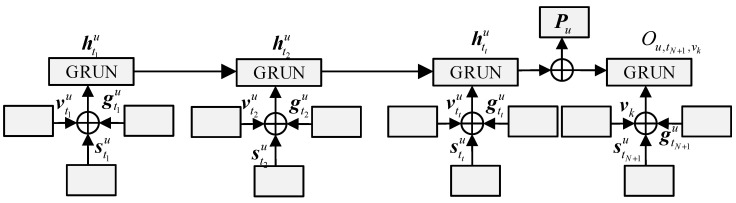
GS-GRUN model structure.

In order to deeply explore the user’s behavior pattern, it must take the context information corresponding to space and time as the entry point and use the scientific modeling of the context relationship corresponding to the personalized space-time. Therefore, it can conduct a more comprehensive analysis of the user’s specific behavior pattern, but the continuous geographical distance and time interval are not paid attention to. From the actual situation, the above information is of key significance to the modeling process and the exploration of user preferences. This section introduces various information about geography and time intervals for the most basic GRUN network, which can provide strong support for the modeling and learning process of users’ personalized spatial and temporal preferences. As shown in Figure 2, the network convolution corresponding to GS-GRUN is described. Further description for all time steps, the output components of its units include the context vectors corresponding to embedding, space, and time. Therefore, the output belongs to the hidden layer vector, which represents the fusion of POIs and spatiotemporal context information. The following are the relevant calculation formulas:

(8) $$\begin{array}{l} z_{t_{k}}^{u}=\delta (U_{z}^{}v_{t_{k}}^{u}+W_{z}^{}h_{t_{k-1}}^{u}+W_{sz}^{}s_{t_{k}}^{u}+W_{gz}^{}g_{t_{k}}^{u}+b_{z}^{})\\ r_{t_{k}}^{u}=\delta (U_{r}^{}v_{t_{k}}^{u}+W_{r}^{}h_{t_{k-1}}^{u}+W_{sr}^{}s_{t_{k}}^{u}+W_{gr}^{}g_{t_{k}}^{u}+b_{r}^{})\\ {\tilde{h}}_{t_{k}}^{u}=\tanh(U_{c}^{}v_{t_{k}}^{u}+W_{sh}^{}s_{t_{k}}^{u}+W_{gh}^{}s_{t_{k}}^{u}+W_{c}^{}(r_{t_{k}}^{u}⊙h_{t_{k-1}}^{u})b_{c}^{})\\ h_{t_{k}}^{u}=(1-z_{t_{k}}^{u})⊙h_{t_{k-1}}^{u}+z_{t_{k}}^{u}⊙{\tilde{h}}_{t_{k}}^{u} \end{array}$$

where, $s_{t_{k}}^{u}$ and $g_{t_{k}}^{u}$ are vector representations of geographical distance and time interval between $v_{t_{k}}^{u}$ and $v_{t_{k}-1}^{u}$, respectively. Additionally, ***U**_z_*, ***U**_r_*, ***U**_c_*, ***W**_z_*, ***W**_r_*, ***W**_sh_*, ***W**_sz,_* and ***W**_gz_* are conversion matrices for the relevant outputs. Moreover, *δ* is transfer function of the POIs. Moreover, $z_{t_{k}}^{u}$ and ${\tilde{h}}_{t_{k}}^{u}$ are the parameters of the spatiotemporal function.

The user’s preferences corresponding to all hidden states can be more comprehensively and deeply learned. It is obvious that if the corresponding training is carried out for all geographical distances and time intervals with continuity, the data sparsity problem that cannot be ignored will occur in this network. Therefore, the values corresponding to geographical distances and time with continuity can be effectively further divided into the range of equal distance intervals, and the correct conversion matrix can be smoothly obtained by means of linear difference.

In addition, the prediction rate corresponding to the POI *v_k_* of user *u* on the time *t_N+1_* can be calculated by using the following formula.

(9) $$O_{u,t_{N+1},v_{k}}={\left(W_{N}^{}h_{t_{N}}^{u}+W_{p}^{}p_{}^{u}\right)}^{T}(W_{v}^{}v_{k}^{}+W_{s}^{}s_{t_{N+1}}^{u}+W_{g}^{}g_{t_{N+1}}^{u})$$

where, *p^u^* is the general expression of user’s *p^u^*, which is mainly able to correctly measure the basic features and long-term preferences of users. $h_{t_{N}}^{u}$ refers to the dynamic representation of users, which scientifically measures the dynamic preferences generated by users within the context of specific time and space. ***W**_N_* and ***W**_P_* are the weight parameters of the output layer. ***W**_v_*, ***W**_s_* and ***W**_g_* are conversion matrices.

In order to make the whole network realize the existence and influence of the loss, that is, in the training process, adjust and change the parameters of the model to minimize the loss function and make the prediction of the model as close as possible to the real value. It is necessary to introduce the optimization function. In deep learning, the loss in the training process is mainly transmitted back to the network through the backpropagation algorithm, so as to update the learnable parameters in the network. Gradient descent is the most popular optimization algorithm, which has the characteristics of fast, strong robustness, and strong flexibility. In the process of exploring the POI recommendation model, this paper specifically uses the Adam optimization algorithm, which is a random objective function optimization algorithm based on first-order gradient and has adaptive estimation.

### 3.4. Model Validation

This study develops an HMG system including 10 kW_p PV, 35 cubical extents biological digestion by a 15 kVA generator, and 1 kW/6 kWh VRFBS at the MG. A suitable protection system is set up to connect the RERs with the distribution grid and the Main Control Panel (MCP). A 3-phase 10 kW inverter is used to connect the 10 kW_p PV plant to the AC network. An engine-generator set feeds the BGP produced by the BGP, which is then synchronized with the grid. A bi-directional converter connects the VRFBS employed as BESS to the AC distribution grid bus. An RP communication network by MODBUS on TCP/IP and RS485 has been applied to control and inspect the MGs on time. In the MG center, the central server is used for monitoring and controlling functions. Table 1 illustrates the procedure flow of the IoT-enabled bidirectional relevance. The energy meters display data from the RERs, the grid, and loads in real time. MODBUS TCP/IP communication protocol is used to connect the energy meters to the Ethernet switchboard. In addition, the BGP generator has been automatically started and stopped via the microcontroller agent that runs into the RP. Afterward, data has been sent to the processor of RP that processes the on-time information and controls the action of the suggested SMG. MATT has been considered a basic messaging communication structure developed for limited equipment using small bandwidth. As a result, MATT has been viewed as a good choice for IoT applications. Sensor nodes can use MATT for sending commands, reading data, and publishing it. As a result, it will be easier for various devices to communicate. This can be done by sending commands for output control, reading the information from the sensors, and publishing them.

In addition, Numerous brokers are available, and Mosquito appears to be one of the ones most commonly utilized here. Message send and receive speeds are affected by the server’s internet speed. The topic supports arbitrary payloads up to 256 MB using 8 bytes (Fixed/Variable) header. With a small pack extent of just two bytes, the individual-byte control view, and the individual-byte pack extent view, MATT can be called the low-bandwidth IoT structure, unlike HTTP, which requires a great deal of bandwidth to function effectively. The HTTP multi-client protocol requires longer execution times since messaging must be sent individually to each user or node each time, while MATT requires a single message to be sent to all linked clients. These benefits explain why the MATT protocol is used in order to establish ICS between various RERs, VRFBS, load, and distribution grids. The present study uses the TS platform for monitoring and controlling the real-time MG information provided by the RP processor. TS is the IoT Middleware which connects to cloud storage or native MATT brokers allowing mass information analysis in a graphical format. This is the unscrew-source IoT usage developed with io-Bridge in 2010 called TS.

## 4. Experiments and Analysis

### 4.1. Data Set

The experimental data are from two public data sets, loc-Gowalla and loc-Brightkite. Gowalla is a social networking site where users can share their location with others by checking in. The loc-Gowalla data set is collected using the Gowalla public API. There are two data sets, one is the data set of 6,442,890 check-in positions of users from February 2009 to October 2010, and the other is the data set of the friends network, which consists of 196,591 nodes and 950,327 links. Brightkite is an LBSN service provider. The check-in location data set is the 4,491,143 check-in records of Brightkite users from April 2008 to October 2010. The friend relationship network is composed of 58,228 nodes and 214,078 links. To help remove the high computational burden, intelligent position sensors are used in this study. It should be noted that smart sensors come in a high variety and would be adopted according to the needs and applications. Such sensors are capable of handling numerous tasks using advanced signal-processing methods. It is proven that intelligent sensors are highly useful for better feature selection and extraction purposes. In our case, intelligent position sensors are used for purifying data and removing outliers.

Processing of normal user data set: remove records with zero location, remove records with less than 10 users, remove records with different points less than 2 corresponding to each user, remove location records with continuous and repeated check-in of each user, and then remove users without check-in records in the friend relationship network. Cold start user data processing: process on the basis of the above normal user data set. Randomly delete the check-in records of users whose check-in records are greater than 8 until the number of check-in records is equal to 8. The specific method is to count the number of visits to each location in the training set. When deleting the user records, delete them according to the location. Once deleting them, the number of visits to the location will be reduced by 1. When the number of visits to the location is equal to 2, the location will not be deleted. Delete the users who have no check-in records in the friend relationship network.

### 4.2. Evaluation Index

In order to accurately measure the recommendation quality of the model proposed in this paper, the most commonly used evaluation indexes, such as *precision*@*k*, *recall*@*k,* and *F*1-Measure, in the recommendation algorithm are used to measure the performance of the proposed method.

For each user *u*, the POI that the user has not checked in is recommended in the data set, and the *precision@k* of the POI recommendation is:

(10) $$precision@k=\frac{\left|P_{}(r)\cap P_{}(u)\right|}{k}$$

where, *P*(*r*) represents the POI set recommended by the proposed method, *P*(*u*) represents the POI set checked in by the user, and *k* is the number of POIs in the recommended POI set.

For each user *u*, the POI that the user has not checked in is recommended in the data set, and the recall@k of POI recommendation is:

(11) $$recall@k=\frac{\left|P_{}(r)\cap P_{}(u)\right|}{\left|P_{}(u)\right|}$$

where, $\left|P_{}(u)\right|$ represents the number of POIs checked in by the user.

*F*1@*k* can better balance the recommendation precision and recall. When the value of *F*1 is closer to 1, the balance between the two is better, and the performance of the POI recommendation algorithm is better. The calculation formula is expressed as:

(12) $$F1@k=\frac{2\cdot (precision@k)\cdot (recall@k)}{recall@k+precision@k}$$

### 4.3. Comparative Experiment and Analysis

**Figure 3:**
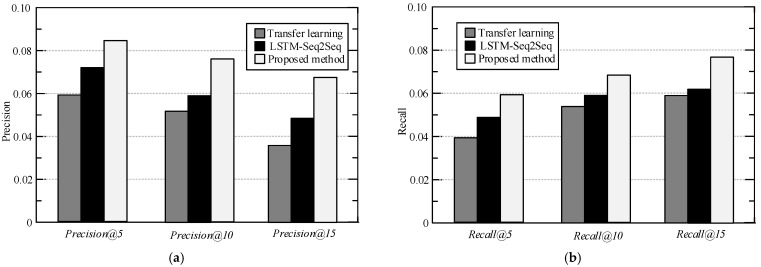
Experimental comparison results on loc-Gowalla data set (**a**) precision (**b**) recall.

**Figure 4:**
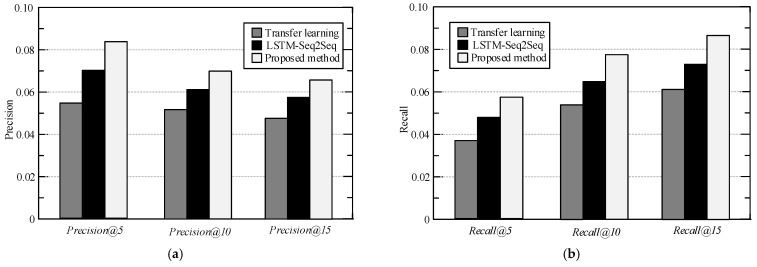
Experimental comparison results on loc-Brightkite data set (**a**) precision (**b**) recall.

**Figure 5:**
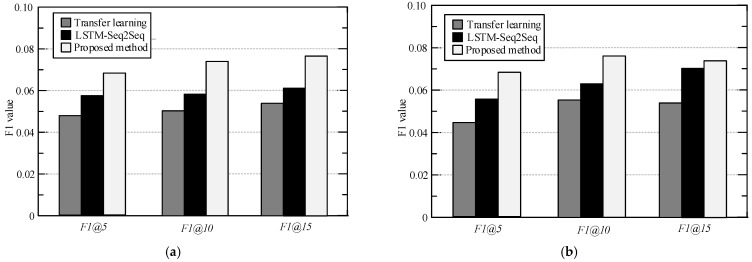
F1 Comparison results of different methods on different data sets. (**a**) loc-Gowalla (**b**) loc-Brightkite.

In order to prove the advantages of the proposed personalized POI recommendation method, under the same experimental conditions, the recommended method in reference [26,27] was compared with the proposed method. *Precision*@*k*, *recall*@*k,* and *F*1@*k* are used to measure the recommendation effect of each model and set the recommended number *K* of POI to 5, 10, and 15, respectively, to exclude the contingency of the experimental results. The experimental results are shown in Figure 3, Figure 4 and Figure 5. It should be noted that methods of “Transfer learning in [26]” and “LSTM-Seq2Seq in [27]” are compared in this part with the same characteristics mentioned in their original papers. For more explanations and details, please refer to these references.

Figure 3 and Figure 4 are the experimental results of each recommendation model on the loc-Brightkite data set and the loc-Gowalla data set. It can be seen from Figure 3 and Figure 4 that the indexes of the proposed method are optimal under all recommended list lengths in the loc-Brightkite data set and the loc-Gowalla data set. When the length of the recommendation list is 15, the precision and the recall of the proposed method on the loc-Gowalla data set are shown in the figures. In all cases, the proposed method could get better results than the others. This is because the proposed method is based on the POI static feature extraction method of symmetric matrix decomposition to capture the geographic location and POI category features in LBSN, and uses the improved CBOW model to extract the semantic features in user comment information. Therefore, the proposed method realizes the hidden vector representation of POI in geographic, category, semantic and temporal feature space, and significantly improves the feature extraction capability of the system. In addition, the user preferences are learned and changed by adaptively selecting relevant check-in activities from the check-in history, so that the proposed method can distinguish the user preferences of different check-in and improve the accuracy of recommendations.

Figure 5 shows the F1 comparison results of different methods on the loc-Brightkite data set and the loc-Gowalla data set. It can be seen from Figure 5 that the indexes of the proposed method are optimal under all recommended list lengths. When the length of the recommended list is 15, the F1 of the proposed method on the loc-Brightkite data set is 0.0783, and the F1 on the loc-Gowalla data set is 0.0748, which is higher than the comparison methods. The first IoT-enabled cost-effective ICS is used to analyze the EMP for various RERs including VRFB-BESSs, BGPs, and PVs in an interconnected network-interactive HMG. This study uses VRFB storage (VRFBS) as BESS since it has many benefits, including scalability and longer cycle life than traditional batteries. Four subsystems make up the suggested MG framework; (1) Cost-effective energy meters in order to collect real-time data for numerous RERs, grids, and loads, (2) MODBUS on TCP/IP and Raspberry-Pi (RP) (Standalone board computer) platforms are used for monitoring and controlling, (3) Utilizing the message alinement telemetry transference (MATT) server and ThingSpeak (TS) Middleware to set up a Cloud-enabled remote monitoring unit (RMU). (4) Measuring BGP capacity and automatically controlling the stop and start of BGP engine generators. HyperText Transfer Protocol (HTTP) is proving to be a widely used server in order to remotely monitor its different applications. This paper, however, adopts MATT to monitor MGs due to its many benefits. MATT is designed to work on low-bandwidth internet, while HTTP is designed for higher-bandwidth internet. IBM designed MATT in 1999 as a lightweight protocol for publishing/subscribing messaging among different devices for IoT-enabled applications. TCP/IP’s application layer uses MATT.

The proposed method uses a symmetric matrix to decompose POI static features and improve the CBOW model to extract semantic features. The proposed method reduces the dimension of the matrix through low-rank approximation, realizes effective utilization of high-dimensional features, captures the potential relationship between static features of POIs, significantly improves the feature extraction ability of the system, and has a better recommendation effect.

## 5. Conclusions

Aiming at the problems that the existing POI recommendation model in social network big data is difficult to extract deep feature information, and the precision and recall are low, a POI recommendation model using deep learning in LBSN big data is proposed. A POI static feature extraction method based on symmetric matrix decomposition is designed to capture the geographical location and POI category features in LBSN, and the semantic features in user comment information are extracted by using the improved CBOW model. The user preferences are learned and changed by adaptively selecting relevant check-in activities from the check-in history, and GSGRUN is used to distinguish the user preferences of different check-in. Experiments show that the proposed method can significantly improve the performance of the POI recommendation system compared with the comparison methods.

The user check-in in the location social network contains rich comment text information and picture information, which contain rich user activity, emotion, and preference characteristics. In the next research, the text features and picture features are extracted by using deep learning technology and integrated into the POI recommendation or user activity recommendation framework. In addition, the time factor is an important attribute of check-in. Considering the different preferences of users in different time periods, it is more consistent with the use rules of applications in real life. In the next study, the time factor will be introduced to study the time-aware POI recommendation method.

## Figures and Tables

**Table 1:** Costs comparing among IoT based RP enabled intelligent controller and LabVIEW enabled intelligent controller.

		Cost (INR)
(a) Smart Controller platform based on RP	HDMI Cable	350
	D-Link DES-1008C 10/100 Mbps switch network	950
	Entire HD IPS Panel Monitor withVGA, HDMI	8750
	RP 3-Layout B	2700
	Entire	12,750
(b) Smart Controller platform based on Lab View	Dedicated Desktop (PG)	30,000
	NI-9871 C Series Interface Module	58,700
	$NI^{*}$cRIO-9075 CompactRIO Controller	144,200
	Entire	232,900

## Data Availability

The data used to support the findings of this study are included within the article.
